# Analyzing the correlation between gastroesophageal reflux disease and anxiety and depression based on ordered logistic regression

**DOI:** 10.1038/s41598-024-57101-2

**Published:** 2024-03-19

**Authors:** Qian Li, Hui Duan, Qiong Wang, Peiwen Dong, Xinxu Zhou, Kaidi Sun, Feng Tang, Xinxin Wang, Lin Lin, Yanchan Long, Xiaobin Sun, Lan Tao

**Affiliations:** The Third People’s Hospital of Chengdu, Chengdu, China

**Keywords:** Gastroesophageal reflux disease, Anxiety, Depression, 24-h esophageal pH monitoring, HADS, Psychology, Gastroenterology

## Abstract

Numerous studies have indicated a connection between psychiatric symptoms, specifically anxiety and depression, and gastroesophageal reflux. However, the precise nature of the link between the severity of gastroesophageal reflux disease and the severity of anxiety and depression remains uncertain. Here, we gathered 24-h pH monitoring data and baseline patient information from a cohort of 518 individuals. Additionally, we evaluated their psychological well-being using the Hospital Anxiety and Depression Scale. The relationship between baseline characteristics and varying degrees of anxiety, depression, and gastroesophageal reflux disease (GERD) was assessed using R software version 4.1.3 and logistic regression models. The findings indicate a statistically significant variation in anxiety levels based on gender, as well as a significant disparity in depression groups when considering age and literacy levels. Kruskal–Wallis test analysis revealed a significant positive correlation between the severity of anxiety and depression and the 24-h pH monitoring results in our patient cohort. As the anxiety and depression levels increased, the rank mean for each examination result also increased. Logistic regression modeling analysis showed that a higher anxiety level was associated with a higher level of GERD. In the presence of mild anxiety, there is a statistically significant association with a higher incidence of GERD with an odds ratio (OR) of 2.64 (95% CI 1.50, 4.64). Similarly, the moderately severe anxiety group also exhibits a causal relationship with an increased GERD incidence, with an OR of 6.84 (95% CI 3.92, 12.17). Additionally, moderate to severe depression is associated with a higher incidence of GERD, with an OR of 2.32 (95% CI 1.23, 4.37). The prevalence of GERD was greater among males compared to females (OR 2.29, 95% CI 1.51–3.49). Additionally, an elevated body mass index (BMI) demonstrated a positive correlation with the susceptibility to GERD (OR 1.07, 95% CI 1.01–1.14). Increasing age may promote the occurrence of GERD in patients. These findings may help to provide a better basis for psychological or pharmacological interventions for GERD patients with psychosomatic symptoms in the future, and provide a reference basis for clinical treatment of the disease.

## Introduction

Gastroesophageal reflux disease (GERD) refers to the backward flow of stomach and duodenal contents into the esophagus, leading to symptoms like acid reflux and heartburn. Additionally, it can give rise to extra-esophageal manifestations, including abdominal distension, hiccups, coughing, and difficulty in swallowing^1^. GERD is a prevalent global clinical issue^2^, with a prevalence of 10–20% in Western nations^3^. In Chinese populations, the incidence of GERD has been steadily increasing due to changing lifestyles and dietary habits^4^. Recurrent disease episodes, frequent doctor visits, prolonged medication usage, and other related factors significantly impair patients' quality of life and overall health. These adverse effects have been well-documented^5^. Moreover, individuals with GERD are particularly susceptible to psychological issues, notably anxiety and depression^6^. The heightened risk of anxiety and depression in GERD patients can be attributed to factors such as acid reflux, heartburn symptoms, and genetic predisposition, further exacerbating the psychosomatic burden^7,8^. This adversely affects the quality of life and prognosis of patients. Anxiety disorders and depression are psychiatric conditions that can manifest as reduced social interaction, negative mood states, and cognitive impairment. They are often accompanied by physical symptoms^9^. Psychoemotional abnormalities can increase sensitivity in the esophagus^6^. This heightened sensitivity contributes to the development of GERD by impacting the brain-gut axis and influencing the neuroendocrine system. Additionally, there are shared genetic factors between gastrointestinal and psychiatric disorders that play a role in GERD pathogenesis^10^. Several studies have explored the impact of psychological factors on GERD^11^. For instance, a study conducted by Rongxin Wang et al. used multivariate analysis to confirm the connection between these psychiatric disorders and the development of reflux esophagitis (RE)^12^. A study conducted by Ye-Seul Lee et al.^13^ on the risk of co-morbidity between psychological disorders and GERD among people in the Korean region similarly showed that psychological disorders and GERD may be inter-related. Psychological symptoms are closely related to quality of life^14^, however, it is frequently encountered that anxiety and depression often go undiagnosed in patients with GERD, primarily because the focus is predominantly on the disease's symptomatic presentation. Consequently, there is a tendency for inadequate management of anxiety and depression in these GERD patients. While numerous prior studies have established a connection between GERD and anxiety and depression^6–8,11,15,16^, none of these studies have explored the potential correlation between the degree of acid reflux, assessed through 24-h pH monitoring, and the severity of anxiety and depression in GERD patients. Another factor deserving consideration is, different dietary habits resulting from different geographic locations may also be responsible for influencing the incidence of GERD in various regions^17–19^. Therefore, it is essential to investigate in western region of China whether there exists a relationship between the severity of anxiety and depression and the severity of gastroesophageal reflux in these patients.

The presence of mucosal injury in the lower esophagus under upper gastrointestinal endoscopy (GI) is diagnostic of RE, which can be further classified into four grades, A, B, C, and D, according to the Los Angeles classification, and GERD can be diagnosed in RE with a Los Angeles classification of grade B and above^20^. However, GERD can also be diagnosed by upper gastrointestinal endoscopy without esophageal mucosal rupture or grade A RE, in patients with typical (or atypical) upper gastrointestinal or extraesophageal symptoms, and with 24-h pH monitoring suggesting the presence of pathologic reflux, in which endoscopic mucosal intactness but 24-h pH monitoring suggesting the presence of pathologic acid reflux can lead to the diagnosis of non-erosive reflux disease (NERD)^21^. Therefore, the diagnosis of GERD severity based on symptoms alone may be inaccurate, and GI endoscopy can only confirm the diagnosis of GERD by observing the integrity of the mucosa, and data on the patient's dynamic reflux are not available. A scholarly study^22^ analyzed the relationship between patients' disease and anxiety and depression based on endoscopic findings, but the specificity of performing endoscopy to diagnose gastroesophageal reflux is not high^1^. At present, the diagnosis of GERD based on pH monitoring results is still a scientific and reliable method^23^. Dynamic esophageal reflux monitoring (24-h pH monitoring) can assess the esophageal reflux load, and by obtaining dynamic reflux data from the patient, it can diagnose whether the patient has GERD and objectively measure the severity of acid reflux in the patient^24^. Therefore, the present study, based on endoscopy, combined with the results of 24-h pH monitoring, analyzes the relationship between the severity of GERD reflux and the anxiety and depression of patients' psychosomatic factors, which will help to better provide the basis for psychological or pharmacological interventions for the diagnosis and treatment of GERD patients accompanied by psychosomatic symptoms, and provide a reference basis for the clinical treatment of the disease.

## Materials and methods

### Inclusion and exclusion criteria

This study retrospectively selected and analyzed 518 patients with typical gastroesophageal reflux symptoms, such as acid reflux and heartburn, who completed endoscopy and 24-h pH monitoring and completed the Hospital Anxiety and Depression Scale (HADS) at a tertiary hospital in Chengdu City from December 2020 to May 2023, which was approved by the Ethics Committee of the Hospital with the approval number 2021-T-16, and the procedures were conducted in accordance with the principles of Declaration of Helsinki. Written informed consent was obtained from all patients before 24-h pH monitoring and fill out the questionnaire. The inclusion criteria were (1) age > 18 years; (2) ability to independently write and sign the informed consent for the examination. The exclusion criteria were (1) patients who failed to complete the full process of 24-h pH monitoring; (2) those who were participating in other clinical trials; (3) those with incomplete investigation data; (4) patients who took drugs that affected esophageal dynamics and acid-suppressing and antacid-suppressing medications during the examination period; (5) those who had a history of other gastrointestinal surgeries, tumors or achalasia of cardia, etc.; (6) patients with a history of other systemic diseases; (7) patients with a combination of serious cardiovascular, cerebrovascular, hepatic, renal and hematopoietic systems and psychiatric disorders; (8) pregnant women and lactating women.

### Hospital Anxiety and Depression Scale (HADS)

Patients completed the demographic data questionnaire and the Hospital Anxiety and Depression Scale (HADS) independently on the examination day. The questionnaires were administered, completed, and collected on-site. Before filling out the questionnaires, a unified instruction was used to explain to the patients the purpose, significance and requirements for filling out the survey, so that the patients could fill out the questionnaires independently according to their actual situation in the month before completing the questionnaires, and they were not allowed to discuss the questionnaires with others or to be influenced by others. For patients with low literacy, the investigator read the questionnaire to the patients item by item and filled it out on their behalf according to their actual situation.

The demographic data questionnaire was designed by the researcher himself, including general information such as age, gender, height, weight, and literacy. Emotional assessments were performed utilizing the HADS, a tool devised by Zigmond and Snaith in 1983 for the evaluation of anxiety and depression in a clinical setting. The scale was utilized for assessing anxiety disorders and depression in non-psychiatric hospital outpatients. A study conducted by Ingvar Bjelland et al.^25^ demonstrated the scale's effectiveness in screening anxiety and depression dimensions, as well as assessing the prevalence of these conditions among non-psychiatric hospital outpatients. The Cronbach's alpha values for HADS-A and HADS-D were 0.83 and 0.82, respectively, indicating good internal consistency. Moreover, both HADS-A and HADS-D exhibited a sensitivity and specificity of 0.8, which is comparable to the General Health Questionnaire (GHQ). The scale comprises 14 items categorized into two domains: 7 questions assess anxiety (A), and 7 questions assess depression (D). Each question is rated on a scale from 0 to 3, resulting in a maximum score of 21 for each domain. The total score for anxiety (A) and depression (D) was calculated independently from the survey responses. Scores for the anxiety and depression subscales were classified as follows: 0–7 for no symptoms, 8–10 for mild symptoms, and 11–21 for moderate to severe symptoms.

### 24-h pH monitoring

All included patients completed upper gastrointestinal endoscopy and 24-h pH monitoring. Upper gastrointestinal endoscopy was performed to exclude organic lesions and anatomical abnormalities in patients and to confirm that patients could complete the 24-h pH monitoring examination. Patients undergoing 24-h pH monitoring were fasted for at least 8 h before the examination until the electrodes were inserted, medications affecting esophageal motility were discontinued 48 h before the examination, and antacid and acid-suppressing medications were discontinued 7 days before the examination to ensure the accuracy of the results. All patients underwent pH monitoring immediately after esophageal manometry. The 24-h pH monitoring examination was performed by inserting the pH electrode into the esophagus through the nasal cavity, placing the distal electrode about 5 cm above the lower esophageal sphincter, and fixing the outer end of the pH catheter to the nose and cheeks in a secure manner, instructing the patients to carry out normal daily activities and diets, and accurately recording the diets, changes in body position, and gastrointestinal symptoms. The patients returned to the monitoring room after 24 h to remove the electrodes, and the staff imported the data recorded by the instruments and the symptoms recorded by the patients into the computer and analyzed the data, the percentage of time the patient was exposed to acid was determined based on the number of refluxes (pH < 4), % time spent in reflux, and number of long refluxes (T ≥ 5 min) in a 24-h pH-monitoring examination to diagnose the presence of pathological reflux in the patient.

### Statistical analysis

Excel 2016 was applied to enter the data, and after checking for errors, the statistical analysis was performed using the software R 4.1.3. The general information was analyzed using descriptive statistics. Measurement data that followed a normal distribution were presented as mean ± standard deviation ($\overline{{\text{x}}}$ ± *s*), while non-normally distributed data were described using the median. Count data were expressed as frequency or composition ratios. One-way analyses were performed using the analysis of variance (normal distribution) or the Kruskal–Wallis test (non-normal data). The frequency or composition ratio was analyzed by Chi-square test. Multifactorial analyses were performed using a two-level multifactorial logistic regression model, and the associations between the independent and dependent variables were analysed using odds ratio (OR), with 95% CI determining the range of values of the OR. The test level α = 0.05, and the difference was considered statistically significant at P < 0.05.

## Results

### The distribution characteristics of baseline information of questionnaire subjects were analyzed

This study retrospectively collected 518 patients who completed 24-h pH monitoring as study subjects. The study population consisted of 224 males (43.24%) and 294 females (56.76%). Most of the study subjects (95.17%) were married. 51.35% of the study subjects had an educational level of junior high school or below. Most of the study subjects did not have lifestyle habits such as drinking alcohol, smoking, coffee, strong tea, etc. 37.64% of the study subjects were infected with *Helicobacter pylori* (HP). Combined with the results of endoscopic mucosal integrity and 24-h pH monitoring results, 275 patients were diagnosed with physiologic reflux, 158 patients with non-erosive reflux disease (NERD), and 85 patients with RE confirmed gastroesophageal reflux. Details of the patients in the study cohort are shown in Table 1.

**Table 1:** Overall patient baseline (N = 518).

Variable	Number of cases/composition ratio
Sex	
Male	224 (43.24%)
Female	294 (56.76%)
Marriage	
Unmarried	25 (4.83%)
Married	493 (95.17%)
Educational level	
Junior high school and below	266 (51.35%)
High school or junior college	117 (22.59%)
University and above	135 (26.06%)
Alcohol consumption	
No	454 (87.64%)
Yes	64 (12.36%)
Smoking	
No	441 (85.14%)
Yes	77 (14.86%)
Coffee	
No	478 (92.28%)
Yes	40 (7.72%)
Strong tea	
No	450 (86.87%)
Yes	68 (13.13%)
Infected with *Helicobacter pylori* (HP)	
Not infected	323 (62.36%)
Infected (including being infected)	195 (37.64%)
Diagnostic subgroup	
Physiologic reflux	275 (53.09%)
NERD	158 (30.5%)
RE	85 (16.41%)

*NERD* non-erosive reflux disease, *RE* reflux esophagitis.

Descriptive analyses of the population characteristics of the study subjects and the results of the 24-h pH monitoring examinations were carried out. The results of the descriptive analysis of the overall population characteristics and examination results of the 518 investigated subjects are given in Table 2. The overall population age was 56.34 ± 12.79 years, the mean body mass index (BMI) was 24.1 kg/m^2^, the median number of refluxes (pH < 4) was 53, the median percentage of total time spent in reflux was 2.5%, the median number of long refluxes (T ≥ 5 min) was 1, and the median DeMeester score was 11.9.

**Table 2:** Descriptive statistics general population characteristics and examination results (N = 518).

Variables	Min	P25	P50	Mean ± SD	P75	Max
Age	20	48	57	56.34 ± 12.79	65	88
Height	1.42	1.56	1.60	1.62 ± 0.08	1.68	1.88
Weight	36.00	52.63	60.00	61.31 ± 11.71	69.38	100.00
BMI	14.19	20.83	23.12	23.41 ± 3.57	25.55	36.63
Number of refluxes (pH < 4)^#^	1	29	53	80.46	93	735
% Time spent in reflux#	0	0.90	2.50	5.5	6.8	72.5
Number of long refluxes (T ≥ 5 min)^#^	0	0	1	2.46	3	34
DeMeester score#	0.40	5.10	11.90	22.18	27.08	256.60

^#^Non-normal data, mean ± standard deviation of non-normal data expressed as mean.

### Analysis of people with different states of anxiety

The lowest score of the total score of the patients' individual anxiety items was 0 and the highest score was 21, with a mean score of 7.56 ± 4.72. According to the Hospital Anxiety and Depression Scale (HADS), 518 study participants were classified into three different anxiety groups: no, mild and moderate to severe anxiety, and the results are shown in Table 3. 266 (0–7 points, 51.35%) of the 518 patients had no anxiety, 89 (8–10 points, 17.18%) had mild anxiety, and 163 (11–21 points, 31.47%) had moderate to severe anxiety. There was no significant difference in the baseline information of patients in the three different anxiety groups (P > 0.05).

**Table 3:** Analysis of people with anxiety in different states (mean ± SD/No. (%), N = 518).

Variables	No anxiety (0–7 points, N = 266)	Mild anxiety (8–10 points, N = 89)	Moderate to severe anxiety (11–21 points, N = 163)	*P*
Age	55.18 ± 13.08	58.45 ± 11.73	57.09 ± 12.73	0.075
Height	1.61 ± 0.08	1.62 ± 0.09	1.62 ± 0.08	0.198
Weight	60.21 ± 11.40	61.61 ± 11.08	62.93 ± 12.41	0.062
BMI	23.16 ± 3.43	23.36 ± 3.07	23.84 ± 4.00	0.159
Sex				0.032
Male	103 (38.72%)	37 (41.57%)	84 (51.53%)	
Female	163 (61.28%)	52 (58.43%)	79 (48.47%)	
Marriage				0.276
Unmarried	17 (6.39%)	3 (3.37%)	5 (3.07%)	
Married	249 (93.61%)	86 (96.63%)	158 (96.93%)	
Educational level				0.197
Junior high school and below	137 (51.50%)	45 (50.56%)	84 (51.53%)	
High school or junior college	51 (19.17%)	21 (23.60%)	45 (27.61%)	
University and above	78 (29.32%)	23 (25.84%)	34 (20.86%)	
Alcohol consumption				0.999
No	233 (87.59%)	78 (87.64%)	143 (87.73%)	
Yes	33 (12.41%)	11 (12.36%)	20 (12.27%)	
Smoking				0.383
No	224 (84.21%)	80 (89.89%)	137 (84.05%)	
Yes	42 (15.79%)	9 (10.11%)	26 (15.95%)	
Coffee				0.877
No	244 (91.73%)	83 (93.26%)	151 (92.64%)	
Yes	22 (8.27%)	6 (6.74%)	12 (7.36%)	
Strong tea				
No	228 (85.71%)	81 (91.01%)	141 (86.50%)	0.434
Yes	38 (14.29%)	8 (8.99%)	22 (13.50%)	
Infected with *Helicobacter pylori* (HP)				0.433
Not infected	173 (65.04%)	53 (59.55%)	97 (59.51%)	
Infected (including being infected)	93 (34.96%)	36 (40.45%)	66 (40.49%)	

Continuous variables are expressed as Mean ± SD and categorical variables are expressed as NO.(%).

### Analysis of people with different states of depression

The lowest score for the total score of the individual depression items of the patients was 0 and the highest score was 19, with a mean score of 6.37 ± 4.39. In this study, we utilized the Hospital Anxiety and Depression Scale (HADS) to assess the psychological well-being of 518 participants. These participants were categorized into three distinct depression groups: none, mild, and moderate to severe depression, as illustrated in Table 4. Among the 518 patients, 297 individuals (57.34%) displayed no signs of anxiety, while 128 individuals (24.71%) scored in the mild anxiety range (8–10 points), and 93 individuals (17.95%) exhibited symptoms indicative of moderate to severe depression (11–21 points). There was no significant difference in the baseline information of the patients in the three different depression groups (P > 0.05).

**Table 4:** Analysis of people with depression in different states (N = 518).

Variables	No depression (0–7 points, N = 297)	Mild depression (8–10 points, N = 128)	Moderate to severe depression (11–21 points, N = 93)	*P*
Age	54.84 ± 13.32	57.96 ± 10.94	58.90 ± 12.89	0.007
Height	1.62 ± 0.08	1.62 ± 0.08	1.61 ± 0.09	0.446
Weight	60.78 ± 11.14	62.65 ± 12.14	61.14 ± 12.84	0.316
BMI	23.18 ± 3.32	23.80 ± 3.82)	23.58 ± 3.96	0.225
Sex				
Male	120 (40.40%)	59 (46.09%)	45 (48.39%)	0.301
Female	177 (59.60%)	69 (53.91%)	48 (51.61%)	
Marriage				0.261
Unmarried	18 (6.06%)	3 (2.34%)	4 (4.30%)	
Married	279 (93.94%)	125 (97.66%)	89 (95.70%)	
Educational level				0.003
Junior high school and below	138 (46.46%)	75 (58.59%)	53 (56.99%)	
High school or junior college	62 (20.88%)	30 (23.44%)	25 (26.88%)	
University and above	97 (32.66%)	23 (17.97%)	15 (16.13%)	
Alcohol consumption				0.758
No	263 (88.55%)	111 (86.72%)	80 (86.02%)	
Yes	34 (11.45%)	17 (13.28%)	13 (13.98%)	
Smoking				0.733
No	256 (86.20%)	107 (83.59%)	78 (83.87%)	
Yes	41 (13.80%)	21 (16.41%)	15 (16.13%)	
Coffee				0.355
No	273 (91.92%)	116 (90.62%)	89 (95.70%)	
Yes	24 (8.08%)	12 (9.38%)	4 (4.30%)	
Strong tea				0.962
No	257 (86.53%)	112 (87.50%)	81 (87.10%)	
Yes	40 (13.47%)	16 (12.50%)	12 (12.90%)	
Infected with *Helicobacter pylori* (HP)				0.063
Not infected	186 (62.63%)	71 (55.47%)	66 (70.97%)	
Infected (including being infected)	111 (37.37%)	57 (44.53%)	27 (29.03%)	

Continuous variables are expressed as mean ± SD and categorical variables are expressed as NO. (%).

### Analysis of people with different states of anxiety based on 24-h pH monitoring results

A normality test of the 24-h pH monitoring results showed that they were all skewed data, therefore, the study was analysed using the Kruskal–Wallis test and Chi-square test (Table 5), one-way analysis of variance (ANOVA) (Table 6) and the median test (Supplementary Table 1). The results of the Kruskal–Wallis test analyses showed that the number of refluxes (pH < 4), the percentage of total time spent in reflux, number of long refluxes (T ≥ 5 min), the DeMeester score, and the number of physiological or pathological refluxes, the results of disease diagnosis by comprehensive gastroscopy were statistically different in the different states of the anxious population (P < 0.05). The results of the ANOVA and the median test were homogeneous with them.

**Table 5:** Analysis of people with different states of anxiety based on 24-h pH monitoring results (rank mean, N = 518).

Variables	No anxiety (0–7points, N = 266)	Mild anxiety (8–10points, N = 89)	Moderate to severe anxiety (11–21 points, N = 163)	*P*
Number of refluxes (pH < 4)	206.79	271.80	338.81	< 0.001
% Time spent in reflux	205.03	276.23	339.25	< 0.001
Number of long refluxes (T ≥ 5 min)	214.19	289.90	316.90	< 0.001
DeMeester score	205.02	269.39	343.01	< 0.001
Oesophageal acid exposure				< 0.001
Physiological reflux	210 (78.95%)	47 (52.81%)	42 (25.77%)	
Pathological reflux	56 (21.05%)	42 (47.19%)	121 (74.23%)	
Diagnostic subgroup				
Physiologic reflux	204 (76.69%)	44 (49.44%)	27 (16.56%)	< 0.001
NERD	35 (13.16%)	29 (32.58%)	94 (57.67%)	
RE	27 (10.15%)	16 (17.98%)	42 (25.77%)	

The results of the Kruskal–Wallis test are expressed as rank averages; *NERD* non-erosive reflux disease, *RE* reflux esophagitis.

**Table 6:** Analysis of people with different states of anxiety based on 24-h pH monitoring results ($\overline{{\text{x}}}$ ± *s*, N = 518).

Variables	No anxiety (0–7points, N = 266)	Mild anxiety (8–10 points, N = 89)	Moderate to severe anxiety (11–21 points, N = 163)	*P*
Number of refluxes (pH < 4)	55.26 ± 64.35	88.82 ± 99.00	117.03 ± 118.79	< 0.001
% Time spent in reflux	3.47 ± 6.81	6.46 ± 8.90	8.29 ± 9.76	< 0.001
Number of long refluxes (T ≥ 5 min)	1.58 ± 3.63	3.17 ± 4.44	3.50 ± 4.36	< 0.001
DeMeester score	14.51 ± 24.86	25.08 ± 33.68	33.10 ± 36.44	< 0.001

Differences between 24-h pH monitoring results in people with different states of anxiety using one-way ANOVA.

### Analysis of people with different states of depression based on 24-h pH monitoring results

A normality test of the 24-h pH monitoring results showed that they were all skewed data, therefore, the study was analysed using the Kruskal–Wallis test and Chi-square test (Table 7), one-way analysis of variance (ANOVA) (Table 8) and the median test (Supplementary Table 2). The results of the Kruskal–Wallis test analysis showed that the number of refluxes (pH < 4), percentage of total time spent in reflux, number of long refluxes((T ≥ 5 min), DeMeester score, and the number of physiological or pathological refluxes, the results of disease diagnosis by comprehensive gastroscopy were statistically different in the depressed population of the different states (P < 0.05). The ANOVA and the median test results were homogeneous with them.

**Table 7:** Analysis of people with different states of depression based on 24-h pH monitoring results (rank mean, N = 518).

Variables	No depression (0–7 points, N = 297)	Mild depression (8–10 points, N = 128)	Moderate to severe depression (11–21 points, N = 93)	*P*
Number of refluxes (pH < 4)	223.76	294.42	325.58	< 0.001
% Time spent in reflux	222.37	300.59	321.51	< 0.001
Number of long refluxes (T ≥ 5 min)	226.35	295.36	316.03	< 0.001
DeMeester score	222.14	299.70	323.47	< 0.001
Oesophageal acid exposure				< 0.001
Physiological reflux	220 (74.07%)	51 (39.84%)	28 (30.11%)	
Pathological reflux	77 (25.93%)	77 (60.16%)	65 (69.89%)	
Diagnostic subgroup				< 0.001
Physiologic reflux	210 (70.71%)	43 (33.59%)	22 (23.66%)	
NERD	51 (17.17%)	56 (43.75%)	51 (54.84%)	
RE	36 (12.12%)	29 (22.66%)	20 (21.50%)	

The results of the Kruskal–Wallis test are expressed as rank averages; *NERD* non-erosive reflux disease, *RE* reflux esophagitis.

**Table 8:** Analysis of people with different states of depression based on 24-h pH monitoring results ($\overline{{\text{x}}}$ ± *s*, N = 518).

Variables	No depression (0–7 points, N = 297)	Mild depression (8–10 points, N = 128)	Moderate to severe depression (11–21 points, N = 93)	*P*
Number of refluxes (pH < 4)	65.87 ± 88.51	95.96 ± 105.82	105.73 ± 89.97	< 0.001
% Time spent in reflux	4.48 ± 8.92	6.74 ± 8.14	7.05 ± 6.93	0.006
Number of long refluxes (T ≥ 5 min)	1.96 ± 4.13	2.97 ± 4.13	3.33 ± 3.79	0.005
DeMeester score	18.92 ± 33.37	26.65 ± 29.54	28.44 ± 26.25	0.004

Differences between 24-h pH monitoring results in depressed populations with different states using one-way ANOVA.

### Logistic regression analysis to model disease influencing factors

**Figure 1:**
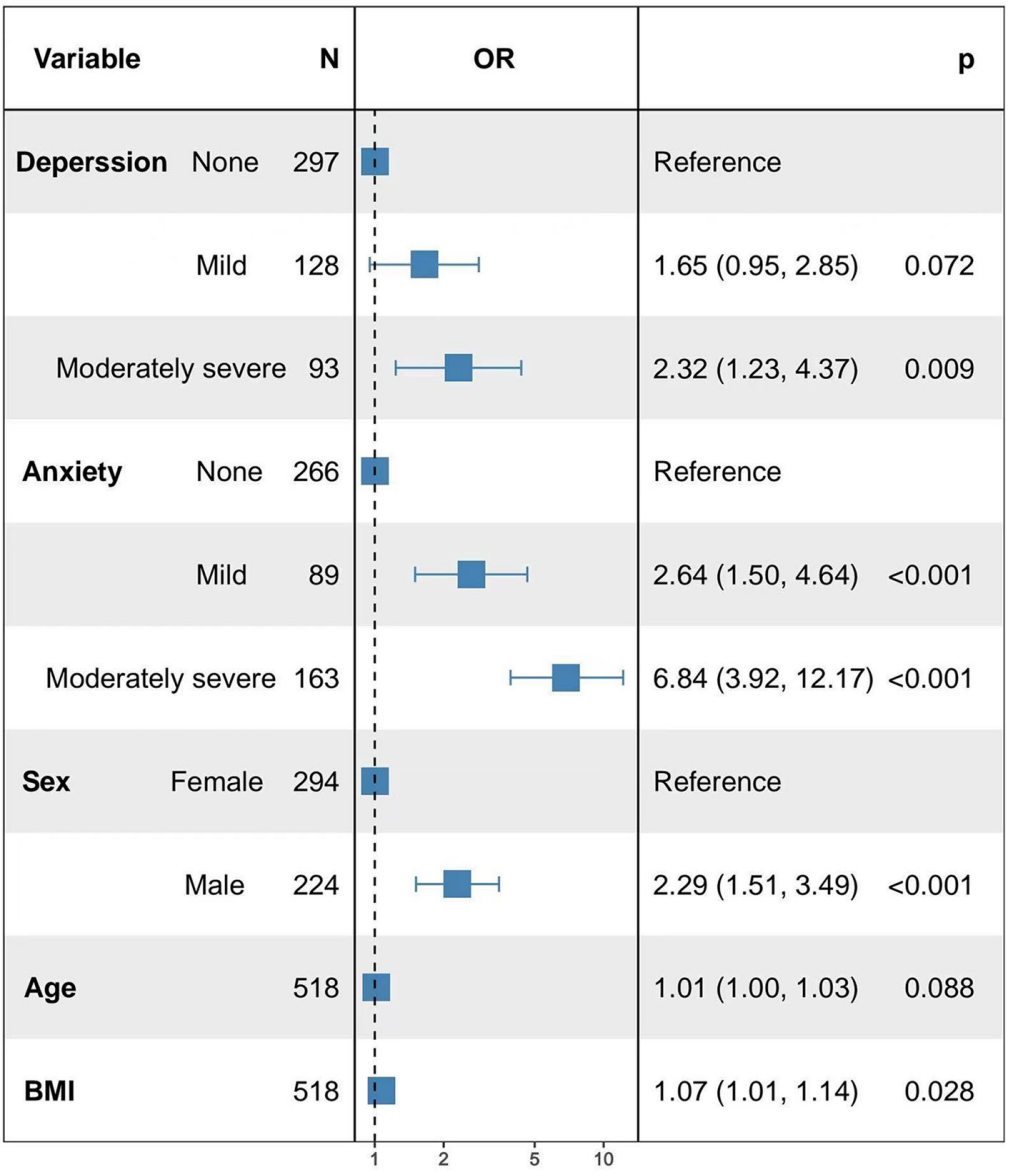
Multifactor logistic regression model.

In this study, we determined the percentage of time of acid exposure and thus diagnosed the presence of pathological reflux in patients based on the results of 24-h pH-monitoring such as the number of refluxes (pH < 4), % time spent in reflux, and the number of long refluxes (T ≥ 5 min). The patient's oesophageal acid exposure status was used as the dependent variable, and anxiety, depression, and baseline information were used as the independent variables to build a logistic regression model, and the final determinants of inclusion are shown in Fig. 1. The model likelihood ratio chi-square value was 147.32, P < 0.01, and the model fit was successful (Supplementary Table 3). The incidence of gastroesophageal reflux disease (GERD) in the mild anxiety group and the moderately severe anxiety group was 2.64 times (95% CI 1.50, 4.64) and 6.84 times (95% CI 3.92, 12.17) higher than that in the no anxiety group, respectively. The incidence of GERD in the moderately severely depressed group was 2.32 times (95% CI 1.23, 4.37) higher than the incidence of GERD in the no-depression group. The incidence of GERD was 2.29 times higher in men than in women (95% CI 1.51, 3.49), and the incidence of GERD rose 1.07 times for every 1 kg/m^2^ rise in BMI (95% CI 1.01, 1.14). In addition, although the results of the model showed that age was not a factor influencing patients' GERD, it suggested that increasing age may promote the occurrence of GERD in patients.

## Discussion

The current study aimed to assess the correlation between the severity of gastroesophageal reflux disease (GERD) and patients' levels of anxiety and depression. This was achieved by integrating data from 24-h pH monitoring and scores obtained from the Hospital Anxiety and Depression Scale (HADS). The 24-h pH monitoring can be the most recommended test for detecting the severity of reflux in patients with GERD and HADS is highly accessible and has multiple advantages such as simplicity of the items, easy operation, easy acceptance by various populations, and availability at the time of the patients' consultation.

The analyses showed that there were between-group differences by gender in the no-anxiety, mild-anxiety, and moderately-severe-anxiety groups, with males having significantly fewer no-anxiety than females in the different states of anxiety. The "gender hypothesis" suggests that women may be more likely to develop mood disorders based on gender experiences while growing up, and the "social factors hypothesis" suggests that women focus differently on life choices, which leads to different expectations, and that they are more likely to develop mood disorders when expectations are not equal to rewards. In general, the differences in mood disorders between genders may be related to the biological basis, coping styles, and socio-cultural factors between genders^26,27^. The Hypothalamic–Pituitary–Adrenocortical (HPA) axis theory suggests that hormone levels fluctuate cyclically over a wider range in females than in males, and that these fluctuations affect the areas of the brain that are involved in regulating mood and behaviour. This makes women more susceptible to stress and therefore at a higher risk of anxiety and depression than men^28^, which was confirmed by the study of Faravelli et al.^27^. Interestingly, however, using logistic regression analysis it was found that the prevalence of GERD was higher in males compared to females (OR 2.29, 95% CI 1.51, 3.49), which is in line with the findings of a community-based cohort study of middle-aged and elderly Australian males, which showed that males had a high prevalence of GERD^15^.

Analyses of the depressed population in different states found intergroup differences in age and literacy between the no-depression group, mild depression, and moderately severe depression, with the older the average age of the patients the more severe the degree of depression. The median age of the respondents in this study was 57 years (interquartile range 48–65 years), and the majority of the respondents were in the perimenopausal period, and it has been demonstrated that women have a higher risk of depression, anxiety, and co-morbid depressive and anxiety disorders prior to menopause which may be related to age-related hormonal changes^27^. The results of the analysis using logistic regression showed that although age is not a direct factor affecting GERD in patients, the analysis found that increasing age may promote the development of GERD in patients (OR 1.01, 95% CI 1.00, 1.03), and some other studies have indicated that there is a positive correlation between GERD and increasing age, and that the prevalence of GERD increases with increasing age^1,29,30^. Therefore, we put it into the final model, suggesting that we should focus on the effect of age on the disease in future disease prevention.

In terms of educational level, among the cohort of depressed patients, the proportion of depressed patients with lower educational level was higher, and previous studies have reported that educational level may lead to different psychological stress by influencing patients' perceptions, and that those with lower educational level are at higher risk of developing GERD^29^. Some studies have shown that genetically predicted higher levels of education are causally associated with a lower risk of depression and that higher levels of education are causally associated with a lower risk of GERD^31^.

In the analysis of 24-h pH monitoring results in patients presenting varying degrees of anxiety and depression, the Kruskal–Wallis test revealed a notable trend: as the severity of anxiety and depression increased, there was a corresponding rise in the ranked means of all recorded outcomes. Statistically significant differences were found between the groups analyzing the number of patients with physiological reflux, reflux esophagitis (RE) and GERD in different anxiety and depression states. The number of patients with pathological acid reflux in the severe anxiety and depression group was significantly higher than that of patients with physiological acid reflux in the same group. Analysis of the data using logistic regression model showed that anxiety increased the incidence of GERD in the mild anxiety group (OR 2.64, 95% CI 1.50, 4.64) and in the moderately severe anxiety group (OR 6.84, 95% CI 3.92, 12.17). Moderate to severe depression increased the incidence of GERD (OR 2.32, 95% CI 1.23, 4.37).The findings of Seyed Alireza Haji Seyed Javadi et al. showed that psychological factors (anxiety and depression) play an important role in the development of GERD^22^. Marco Losa et al. showed that anxiety and depression play an important role in non-erosive reflux disease (NERD) in addition to reflux hypersensitivity and functional heartburn patients^6^.

We also found that body mass index (BMI) was another risk factor influencing the occurrence of GERD (OR 1.07, 95% CI 1.01, 1.14). Increased BMI was positively associated with the risk of GERD which has been confirmed in other studies^1,32,33^. In this study, we did not find any association between strong tea, smoking, coffee, alcohol consumption, *Helicobacter pylori* (HP), etc. and the severity of GERD, which is consistent with a study by Rongxin Wang et al. who found that the habit of drinking strong tea was positively associated with RE, but not with the severity of RE^12^. This may be related to the type of tea, different concentrations, etc., which may also be due to multiple regression analyses that exclude multifactor interactions. This may be due to differences in the socio-demographic status of the study participants, sample size, data collection and assessment methods, and further research is needed.

This study has some limitations. This study is a single-centre study and most of the data came from the subjective self-reports of the respondents, there may be some recall bias during the information collection process, we omitted other types of highly probable variables from the analysis including strong tea, HP, etc., so extrapolation of the results needs to be further confirmed.

In conclusion, there is a certain correlation between GERD and anxiety and depression, which provides theoretical references for individuals and clinical workers to focus on patients' psychological emotions when treating GERD. In subsequent studies, we will also explore a series of psychological adjustment methods to be used in patients with GERD, with a view to alleviating the various symptoms and severity of the disease and improving the quality of life of patients.

### Supplementary Information

Supplementary Information.

## Data Availability

The datasets used and analyzed during the current study available from the corresponding author on reasonable request.
