# Supplementary material for: Analyzing the correlation between gastroesophageal reflux disease and anxiety and depression based on ordered logistic regression
Source: Sci Rep. 2024 Mar 19;14:6594. doi: 10.1038/s41598-024-57101-2 (PMC10951219; doi:10.1038/s41598-024-57101-2)
Supplement: Supplementary file 1 — Supplementary Information. [file 41598_2024_57101_MOESM1_ESM.doc]

| Supplementary Table 1. Analysis of people with different states of anxiety based on 24-hour pH monitoring results（N=518) | | | | | |
| --- | --- | --- | --- | --- | --- |
| Variables（median） | | No anxiety  （0~7points，N=266） | Mild anxiety  （8~10points，N=89） | Moderate to severe anxiety （11~21points，N=163） | *P* |
| Number of refluxes (pH<4)（53.000） | |  |  |  | <0.001 |
|  | >median | 92 | 45 | 121 |  |
|  | ≤median | 174 | 44 | 42 |  |
| % Time spent in reflux（2.500） | |  |  |  | <0.001 |
|  | >median | 83 | 47 | 127 |  |
|  | ≤median | 183 | 42 | 36 |  |
| Number of long refluxes (T≥5min)（1.000） | |  |  |  | <0.001 |
|  | >median | 69 | 45 | 95 |  |
|  | ≤median | 197 | 44 | 68 |  |
| DeMeester score（11.900） | |  |  |  | <0.001 |
|  | >median | 81 | 47 | 130 |  |
|  | ≤median | 185 | 42 | 33 |  |
| Oesophageal acid exposure | |  |  |  | <0.001 |
|  | Physiological reflux | 210 (78.95%) | 47 (52.81%) | 42 (25.77%) |  |
|  | Pathological reflux | 56 (21.05%) | 42 (47.19%) | 121 (74.23%) |  |
| Diagnostic subgroup | |  |  |  |  |
|  | Physiologic reflux | 204（76.69%） | 44（49.44%） | 27（16.56%） | <0.001 |
|  | NERD | 35（13.16%） | 29（32.58%） | 94（57.67%） |  |
|  | RE | 27（10.15%） | 16（17.98%） | 42（25.77%） |  |

NERD: non-erosive reflux disease; RE: reflux esophagitis.

Supplementary Table 2. Analysis of people with different states of depression based on 24-hour pH monitorin results（N=518)

| Variables（median） | | No depression  （0~7points，N=297） | Mild depression  (8~10points，N=128） | Moderate to severe depression  （11~21points，N=93） | *P* |
| --- | --- | --- | --- | --- | --- |
| Number of refluxes (pH<4)（53.000） | |  |  |  | <0.001 |
|  | >median | 115 | 79 | 64 |  |
|  | ≤median | 182 | 49 | 29 |  |
| % Time spent in reflux（2.500） | |  |  |  | <0.001 |
|  | >median | 110 | 79 | 68 |  |
|  | ≤median | 187 | 49 | 25 |  |
| Number of long refluxes (T≥5min)（1.000） | |  |  |  | <0.001 |
|  | >median | 91 | 66 | 52 |  |
|  | ≤median | 206 | 62 | 41 |  |
| DeMeester score（11.900） | |  |  |  | <0.001 |
|  | >median | 106 | 82 | 70 |  |
|  | ≤median | 191 | 46 | 23 |  |
| Oesophageal acid exposure | |  |  |  | <0.001 |
|  | Physiological reflux | 220 (74.07%) | 51 (39.84%) | 28 (30.11%) |  |
|  | Pathological reflux | 77 (25.93%) | 77 (60.16%) | 65 (69.89%) |  |
| Diagnostic subgroup | |  |  |  | <0.001 |
|  | Physiologic reflux | 210（70.71%） | 43（33.59%） | 22（23.66%） |  |
|  | NERD | 51（17.17%） | 56（43.75%） | 51（54.84%） |  |
|  | RE | 36（12.12%） | 29（22.66%） | 20（21.50%） |  |

NERD: non-erosive reflux disease; RE: reflux esophagitis.

Supplementary Table 3. Model fitting parameters

| Likelihood ratio chi-square | AIC | BIC | P |
| --- | --- | --- | --- |
| 147.32 | 577.29 | 611.29 | ＜0.05 |
